# Prevalence of Acne and Its Impact on Quality of Life, Social Appearance Anxiety and Treatment Practices Among Young Adults

**DOI:** 10.1111/jocd.70654

**Published:** 2026-01-05

**Authors:** Tajin Ahmed Jisa, Md. Tahalil Islam Rahat, Most. Shermin Akter Sumi, Nourin Sultana, Jibon Kumar Sarker, Mifta Nurejannath, Md. Kaderi Kibria

**Affiliations:** ^1^ Department of Statistics Hajee Mohammad Danesh Science and Technology University Dinajpur Bangladesh; ^2^ Department of Statistics University of Rajshahi Rajshahi Bangladesh

**Keywords:** acne vulgaris, Bangladesh, prevalence, quality of life, social appearance anxiety, young adults

## Abstract

**Background:**

Acne vulgaris is a common dermatological condition among young adults. It can cause psychosocial consequences that go beyond its physical effects.

**Aims:**

This study aims to assess acne prevalence and its impact on quality of life, social appearance anxiety, and treatment practices among university students in northern Bangladesh.

**Methods:**

A cross‐sectional survey was conducted among 1067 students to assess acne prevalence and severity (Global Acne Grading System, GAGS). Quality of life, social appearance anxiety, and psychological distress were measured using the Dermatology Life Quality Index (DLQI), Social Appearance Anxiety Scale (SAAS), and Hospital Anxiety and Depression Scale (HADS). Sociodemographic, lifestyle, and familial factors were also evaluated. Descriptive statistics, chi‐square tests, and binary logistic regression were used to identify significant associations and predictors.

**Results:**

Acne was reported by 47.6% of participants. Among those affected (*n* = 465), 47.96% had mild acne, 44.95% had moderate acne, 6.02% had severe acne, and 1.08% had very severe acne. Increasing acne severity was significantly associated with poorer quality of life (*p* < 0.001) and higher social appearance anxiety (*p* = 0.013). Logistic regression identified several independent predictors of acne, including gender (*p* = 0.008), residence (*p* ≤ 0.033), middle‐income status (*p* = 0.040), belief that diet affects acne (*p* < 0.001), smoking (*p* = 0.023), alcohol consumption (*p* = 0.001), and family history of acne (*p* < 0.001). Higher DLQI scores were also significantly associated with acne (*p* = 0.002), whereas SAAS and HADS were not independently associated. Among affected individuals, 33.7% consulted a dermatologist, while others relied on self‐medication (29.9%), home remedies (30.8%), or combined approaches (23.7%).

**Conclusions:**

Acne vulgaris is highly prevalent among young adults in northern Bangladesh and substantially impacts quality of life and social appearance. Its development involves a multifactorial interplay of genetic, lifestyle, and psychosocial factors, emphasizing the need for evidence‐based management, lifestyle interventions, and psychosocial support in student health programs. Future research should examine causal pathways and guide comprehensive prevention and treatment strategies.

**Abbreviations**
BRUR: Begum Rokeya University
DLQI: Dermatology Life Quality Index
DMC: Dinajpur Medical College
GAGS: Global Acne Grading System
HADS: Hospital Anxiety and Depression Scale
HSTU: Hajee Mohammad Danesh Science and Technology University
MICE: Multiple Imputation by Chained Equations
RMC: Rangpur Medical College
SAAS: social appearance anxiety

## 1 Introduction

Acne vulgaris is a chronic inflammatory disorder of the pilosebaceous unit that is characterized by the presence of papules, pustules, nodules, and in severe cases, permanent scarring. It is one of the most common dermatological conditions treated worldwide. It is considered the eighth most prevalent disease globally and affects approximately 9.4% of the population [1, 2, 3, 4]. Although acne most frequently occurs during adolescence, it can persist into adulthood. The condition is increasingly recognized as having significant physical, psychological, and social consequences [5]. Beyond its dermatological manifestations, acne vulgaris has a profound impact on mental health and quality of life. Individuals with acne often report feelings of embarrassment, low self‐esteem, social withdrawal, depression, anxiety, and stress, particularly when lesions recur or lead to visible scarring [6, 7]. Studies have shown that acne‐related psychological distress may extend to body image dissatisfaction, relationship difficulties, academic underperformance, and even, in extreme cases, suicidal ideation [7, 8, 9]. In fact, an earlier dermatological study in the 1990s highlighted that nearly half of reported suicide cases among dermatology patients were linked to acne [8]. Unlike acute conditions, acne follows a fluctuating course in severity and distribution, often requiring prolonged treatment over several months or years [10, 11].

The burden of acne varies across populations and settings. Among medical students, prevalence rates have been reported to range from 34.3% to 97.9% [12]. In Saudi Arabia, 78.5% of health science students reported acne. More than half of them resorted to self‐medication without seeking medical consultation [13]. Similarly, studies from Syria and Malaysia have reported prevalence rates of 34.7% and 75.8%, respectively, with notable gender differences such as female students experiencing significantly greater impairment in quality of life and higher rates of sleep disturbances compared to males [14, 15]. These findings underscore the widespread nature of acne among young adults and its psychosocial implications. Self‐medication is a common practice in acne management. It includes the use of over‐the‐counter medications, herbal and traditional remedies, or dietary supplements [13, 16]. Among university and medical students, the tendency toward self‐treatment is influenced by easy access to information and advice from peers or seniors. Exposure to clinical knowledge and the ready availability of pharmaceutical samples and non‐prescription drugs also contribute to this behavior. While self‐medication may provide temporary relief, unsupervised and inappropriate use of treatments carries risks of delayed diagnosis, medication misuse, side effects, and poor clinical outcomes.

In Bangladesh, acne is a common dermatological concern among young adults. Most research has focused on its clinical aspects, while few studies have addressed its prevalence, psychosocial burden and treatment practices in student populations. Few studies have explored the relationship between acne, quality of life and psychological outcomes such as social appearance anxiety. Understanding these dimensions is crucial because young adults are particularly vulnerable to the psychosocial impact of visible skin disorders which can affect academic performance, interpersonal relationships and overall well‐being. Therefore, this study was designed to investigate the prevalence of acne and assess its impact on quality of life, social appearance anxiety and treatment practices among young adults in Bangladesh. By highlighting psychosocial and behavioral dimensions, the findings aim to inform evidence‐based awareness initiatives, promote responsible treatment practices and support comprehensive management strategies for this prevalent condition.

## 2 Materials and Methods

### 2.1 Study Design and Population Settings

A cross‐sectional study was conducted among undergraduate students and postgraduate students enrolled in four academic institutions located in the Rangpur division of northern Bangladesh named Hajee Mohammad Danesh Science and Technology University (HSTU), Begum Rokeya University (BRUR), Rangpur Medical College (RMC), and Dinajpur Medical College (DMC). Data were collected from January to March 2025 using a self‐structured questionnaire. A total of 1067 students participated in the study.

### 2.2 Inclusion and Exclusion Criteria for Participant Selection

The target population consisted of students enrolled in universities within the Rangpur Division of Bangladesh. Eligible participants were young adults of either sex, currently registered as undergraduate or postgraduate students at any level of study from first year through final year. Individuals who were not enrolled in a university during the study period were excluded from participation.

### 2.3 Sample Size Determination and Sampling Technique

The sample size was determined using Cochran's formula for prevalence studies ($n=Z^{2}p\left(1-p\right)/e^{2}$) with a *Z*‐score of 1.96 for a 95% confidence level. The anticipated prevalence (*p*) was conservatively set at 0.50 to maximize precision and the margin of error (*e*) was 0.03. A total of 1067 students were recruited. After data cleaning and exclusion of incomplete responses, the final analytic sample included 976 participants. A multi‐stage sampling approach was employed to select study participants. In the first stage, four higher educational institutions located in the Rangpur Division were purposively selected named HSTU, BRUR, RMC and DMC. In the second stage, faculties within each institution were stratified according to discipline. From each stratum, departments were randomly chosen to ensure representation across diverse academic backgrounds. In the third stage, academic years within the selected departments were identified and students were randomly approached to participate in the study. This multi‐stage strategy facilitated the inclusion of participants from different universities, disciplines and academic levels, thereby enhancing the representativeness of the sample.

### 2.4 Questionnaire Development, Data Collection and Processing

A self‐administered questionnaire was developed based on an extensive review of relevant literature to comprehensively capture information on: (i) sociodemographic characteristics, (ii) lifestyle factors, (iii) acne severity which was assessed using validated instruments Global Acne Grading System (GAGS) [3], (iv) quality of life which was assessed by the Dermatology Life Quality Index (DLQI) [17], (v) social appearance anxiety which as measured with the Social Appearance Anxiety Scale (SAAS) [18], (vi) psychological impact which was assessed through the Hospital Anxiety and Depression Scale (HADS) [19] and (vii) acne treatment and management practices. Reliability analysis for the DLQI, SAAS, and HADS scales provided Cronbach's alpha values of 0.82, 0.88, and 0.83 respectively. A pilot test was conducted to evaluate the clarity and comprehensibility of the questionnaire. Following the pilot phase, minor revisions were made based on participant feedback. The final version of the questionnaire was translated from English to the local language (Bangla), while the English version is provided in Appendix S1. The final questionnaire was subsequently reviewed and validated by two experts in public health and epidemiology. Printed questionnaires were distributed to eligible participants who were allotted adequate time to complete them. Before data collection commenced, the objectives and significance of the study were clearly explained and written informed consent was obtained. Anonymity and confidentiality were ensured throughout the process. Data quality was maintained through systematic checks for completeness with missing values addressed using Multiple Imputation by Chained Equations (MICE) to reduce potential bias. Multicollinearity among predictor variables was assessed using a correlation matrix to confirm variable independence for subsequent statistical analyses. Duplicate entries were identified through dataset screening and excluded.

### 2.5 Variable Measures

#### 2.5.1 Outcome Variable

The primary outcome of interest was the presence of acne, recorded as a binary response (yes/no). Among those reporting acne, clinical severity was assessed using the GAGS, which is a validated scoring tool that integrates lesion type and anatomical distribution. GAGS scores were categorized as none (0), mild (1–18), moderate (19–30), severe (31–38), and very severe (≥ 39).

#### 2.5.2 Predictor Variables

Explanatory variables were selected to capture sociodemographic, lifestyle and psychosocial domains. Sociodemographic characteristics included age (< 20, 20–23, > 23 years), gender, marital status, residence (urban, suburban, or rural), socioeconomic status (poor, middle, rich), institution, academic year, and family history of acne. Lifestyle factors covered habitual sleep duration (< 5, 5–7, 7–9, > 9 h), smoking, alcohol use and dietary perceptions regarding acne. Psychosocial indicators were measured using validated instruments such as the DLQI for quality of life, the HADS for psychological distress and the SAAS for social appearance anxiety (see Appendix S2 for details). Additionally, information on acne treatment and management practices was collected.

### 2.6 Statistical Analysis

Descriptive statistics were computed for all categorical variables and presented as frequencies and percentages. The normality of continuous variables was evaluated using the Shapiro–Wilk test. Associations of acne prevalence with other factors were assessed using the chi‐square (*χ* ^2^) test. Variables showing a significance level of *p* < 0.05 in the bivariate analysis were subsequently included in a binary logistic regression model to identify independent predictors of acne vulgaris. Adjusted odds ratios (ORs) with 95% confidence intervals (CIs) were reported. A *p*‐value < 0.05 was considered indicative of statistical significance. Data analysis was executed utilizing SPSS (version 26) and R software (version 4.0.2). This study was reported in accordance with the Strengthening the Reporting of Observational Studies in Epidemiology (STROBE) guidelines.

## 3 Results

### 3.1 Socio‐Demographic Characteristics of the Study Participants

A total of 976 respondents participated in the study. The age distribution revealed that the majority were between 20 and 23 years old (69.1%), followed by individuals aged over 23 years (21.8%) and those under 20 years (9.1%), suggesting a predominantly undergraduate population. In terms of gender, 60.7% of the participants were male, while 39.3% were female. Regarding marital status, most of the respondents were unmarried (82.5%), whereas only 17.5% were married. When assessing their living environment, 46.3% reported living in rural areas, 38.9% in urban areas, and 14.8% in suburban regions, providing a diverse representation of environmental contexts. The respondents represented students from four different institutes: HSTU (40.9%), BRUR (23.8%), DMC (19.0%) and RMC (16.3%). In terms of educational level, the largest proportion were in their first year (51.7%), followed by second year (19.2%), third year (17.3%), fourth year (8.1%), graduated (2.3%), and fifth year or master's level (1.1%). Analysis of socioeconomic status showed that 74.3% of participants came from middle‐income families, 13.0% from rich families, and 12.7% from poor backgrounds. Regarding sleeping habits, over half (53.9%) reported sleeping 5–7 h, while 35.2% slept 7–9 h. Only a small proportion reported sleeping less than 5 h (5.3%) or more than 9 h (5.5%), which may have implications for lifestyle and health behaviors. A notable 62.9% of participants reported a positive family history of acne, suggesting a potential genetic or hereditary component, a possible genetic predisposition, whereas 37.1% reported no such family history (see Table 1).

**TABLE 1:** Sociodemographic characteristics of the study participants (*n* = 976).

Variables	*n* (%)	Variables	*n* (%)
Age		Institute	
< 20	89 (9.1)	Brur	232 (23.8)
20–23	674 (69.1)	Rmc	159 (16.3)
> 23	213	Dmc	186 (19.0)
Gender		Hstu	399 (40.9)
Male	592 (60.7)	Education	
Female	384 (39.3)	First year	505 (51.7)
Marital status		Second year	187 (19.2)
Unmarried	805 (82.5)	Third year	169 (17.3)
Married	171 (17.5)	Fourth year	79 (8.1)
Living environment		Graduated	22 (2.3)
Urban	380 (38.9)	Fifth year/Masters	11 (1.1)
Rural	452 (46.3)	Socioeconomic status	
Suburban	144 (14.8)	Poor	124 (12.7)
Sleeping hour		Middle	725 (74.3)
< 5 h	52 (5.3)	Rich	127 (13.0)
5–7 h	526 (53.9)	Family history of acne	
7–9 h	344 (35.2)	Yes	362 (62.9)
> 9 h	54 (5.5)	No	614 (37.1)

### 3.2 Lifestyle Factors of the Study Participants

**FIGURE 1:**
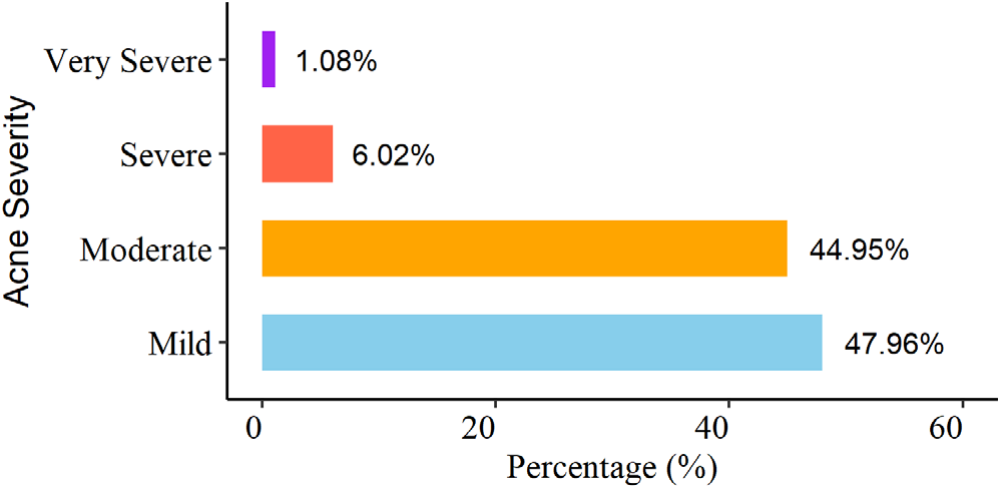
Acne severity levels measured by the Global Acne Grading System (GAGS) whereas the x‐axis indicates the acne severity and the y‐axis indicates the corresponding percentages.

Table S1 presents the assessment of acne‐related lifestyle factors and mental health parameters among the study participants. Nearly half of the respondents (47.6%) reported experiencing acne, while 52.4% did not. Regarding lifestyle factors, 18.6% of participants were smokers and 6.9% consumed alcohol. A majority of participants (58.2%) indicated that their dietary habits influenced acne occurrence. Based on the GAGS, 51.5% of participants had mild acne, 40.8% moderate, 6.5% severe, and 1.2% very severe. The impact of acne on quality of life, as measured by the DLQI, varied, with most participants reporting small (34.4%) to moderate effects (29.7%), while 23.2% experienced a very large effect and 6.2% reported an extremely large effect. SAAS scores among participants with acne indicated that 38.7% experienced mild anxiety, 51.0% moderate, and 10.3% severe anxiety. In contrast, mental health indicators assessed using the HADS revealed that most participants exhibited mild (65.4%) or moderate (26.1%) psychological symptoms, while only 2.8% had severe levels. Normal scores accounted for 5.7% of the sample, indicating that emotional distress was present but generally not severe in most respondents. Among the 976 participants, 465 were reporting acne, severity was evaluated using the Global Acne Grading System (GAGS). Most individuals exhibited mild (47.96%) or moderate (44.95%) acne, whereas a smaller proportion presented with severe (6.02%) or very severe (1.08%) forms of the condition (see Figure 1).

### 3.3 Associated Factors of Acne Prevalence

Among 976 students, 465 (47.6%) reported acne and 511 (52.4%) were acne‐free (Table S2). Age was significantly associated with acne (*p* = 0.001); 31.4% of participants older than 23 years had acne compared to 13.1% without, while students aged 20–23 years were more likely to be acne‐free (76.9%). Gender also showed a strong association (*p* = 0.001) with acne more common in females (45.6%) than males (33.7%). Marital status was significant (*p* = 0.001) as 22.1% of married students were in the acne group compared to 13.3% in the non‐acne group. Educational level was also associated (*p* = 0.001) with higher prevalence among third‐ and fourth‐year students, while first‐years were predominantly acne‐free (58.1%). Family history emerged as a strong predictor (*p* = 0.001) reported by 60.4% of students with acne compared to 15.9% without. Lifestyle behaviors were significantly linked to acne: smoking (25.2% vs. 12.7%, *p* = 0.001), alcohol consumption (11.4% vs. 2.7%, *p* = 0.001) and perceived dietary influence (75.7% vs. 42.3%, *p* = 0.001) were more frequent among students with acne. Table 2 presents the associations between acne severity and psychosocial outcomes including quality of life, social appearance anxiety and psychological distress. Acne severity showed a significant association with quality of life (*χ* ^2^ = 129.37, *p* < 0.001) and with social appearance anxiety (*χ* ^2^ = 8.65, *p* = 0.013), indicating that students with more severe acne experienced greater impairment and higher appearance‐related anxiety. In contrast, no significant association was observed between acne and psychological distress as measured by the HADS.

**TABLE 2:** Association of acne severity with DLQI, SAAS, HADS.

Variable (Impact categories)	No acne *n* (%)	Acne *n* (%)	Chi‐square (χ^2^)	*p*
DLQI			129.37 (4)	< 0.001
No effect	185 (36.2)	30 (6.5)		
Small effect	112 (21.9)	160 (34.4)		
Moderate effect	90 (17.6)	138 (29.7)		
Very large effect	101 (19.8)	108 (23.2)		
Extremely large effect	23 (4.5)	29 (6.2)		
SAAS			8.65 (2)	0.013
Mild	240 (47.0)	180 (38.7)		
Moderate	213 (41.7)	237 (51.0)		
Severe	58 (11.4)	48 (10.3)		
HADS			1.418 (3)	0.701
Normal	33 (6.5)	23 (4.9)		
Mild	329 (64.4)	309 (66.5)		
Moderate	136 (26.6)	119 (25.6)		
Severe	13 (2.5)	14 (3.0)		

### 3.4 Identification of Risk Factors Related to Acne

Binary logistic regression analysis revealed several sociodemographic and lifestyle factors significantly associated with acne vulgaris. In Table 3, male participants had lower odds of acne compared to females (OR = 0.65; 95% CI, 0.47–0.89; *p* = 0.008), while living in rural (OR = 0.51; 95% CI, 0.36–0.73; *p* < 0.001) or suburban areas (OR = 0.59; 95% CI, 0.36–0.96; *p* = 0.033) reduced the likelihood of acne. Middle‐income status was associated with lower odds of acne compared to poor income (OR = 0.61; 95% CI, 0.38–0.98; *p* = 0.040). Participants who believed that diet affected acne had higher odds of acne (OR = 3.47; 95% CI, 2.51–4.78; *p* < 0.001), while non‐smokers (OR = 1.69; 95% CI, 1.08–2.64; *p* = 0.023) and non‐alcohol consumers (OR = 3.48; 95% CI, 1.66–5.30; *p* = 0.001) were also more likely to report acne. Notably, a family history of acne was the strongest predictor, with substantially increased odds of acne (OR = 4.25; 95% CI, 2.45–6.78; *p* < 0.001). When psychological factors were included, higher DLQI scores remained significantly associated with acne (OR = 1.68; 95% CI, 1.22–2.33; *p* = 0.002), indicating that impaired quality of life is an independent risk factor (Table S3). Similarly, Tables S4 and S5 show that male gender, rural/suburban residence, middle‐income status, dietary perceptions, smoking, alcohol consumption and family history consistently remained significant predictors when SAAS and HADS were added to the models. However, SAAS (OR for moderate = 1.14, *p* = 0.450; OR for severe = 1.36, *p* = 0.236) and HADS (OR for mild = 1.45, *p* = 0.290; OR for moderate = 1.32, *p* = 0.458; OR for severe = 1.87, *p* = 0.268) were not independently associated with acne, suggesting that social appearance anxiety and general anxiety/depression symptoms do not contribute significantly beyond demographic and lifestyle factors.

**TABLE 3:** Binary logistic regression analysis of sociodemographic and lifestyle factors associated with acne vulgaris.

Predictor variable	Odds ratio (OR)	*p*	95% CI (lower – upper)
Age of the respondent (Ref: < 20 years)			
20–23 years	0.671	0.146	0.392–1.149
> 23 years	1.392	0.371	0.674–2.877
Gender (Ref: Female)			
Male	0.647	0.008	0.468–0.894
Living environment (Ref: Urban)			
Rural	0.513	0.000	0.361–0.730
Suburban	0.587	0.033	0.359–0.958
Marital status (Ref: Unmarried)			
Married	1.156	0.547	0.722–1.851
Education (Ref: First year)			
Second year	1.070	0.924	0.267–4.294
Third year	0.594	0.485	0.138–2.565
Fourth year	0.471	0.381	0.088–2.535
Graduate	0.927	0.916	0.227–3.786
Fifth year	0.757	0.695	0.189–3.043
Family income (Ref: Poor)			
Middle income	0.607	0.040	0.378–0.976
Upper income	0.618	0.141	0.326–1.173
Belief that diet affects acne (Ref: No)			
Yes	3.468	0.000	2.514–4.784
Sleep duration (Ref: < 5 h)			
5–7 h	1.96	0.636	0.570–2.508
7–9 h	0.982	0.961	0.461–1.933
> 9 h	0.750	0.552	0.291–2.642
Smoking status (Ref: No)			
Yes	1.685	0.023	1.074–2.642
Alcohol consumption (Ref: No)			
Yes	3.479	0.001	1.657–5.301
Family history of acne (Ref: No)			
Yes	4.248	0.000	2.445–6.785

### 3.5 Treatment and Management

**FIGURE 2:**
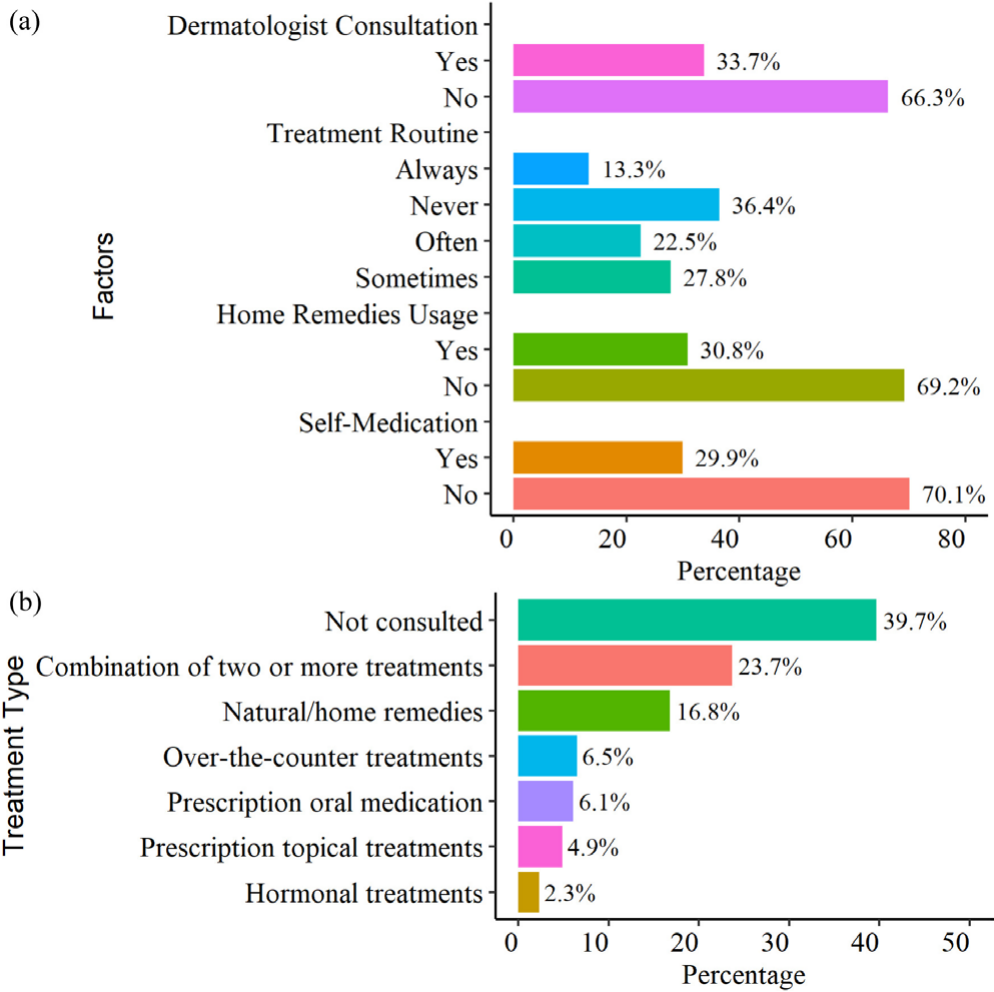
Bar plot. (a) acne treatment and management practices and (b) distribution of acne treatment strategies among study participants.

Among the study participants, 33.7% consulted a dermatologist for acne management. The majority of the participants, 66.3%, did not seek professional care (see Figure 2a). Regarding treatment adherence, 13.3% always followed a regimen, 22.5% often followed it, 27.8% sometimes followed it, and 36.4% never adhered consistently. Home remedies were used by 30.8% of students, while 69.2% did not use any. Self‐medication was reported by 29.9% students. As shown in Figure 2b, the most common approach was no treatment (39.7%), followed by natural or home remedies (16.8%). Pharmacological treatments were less frequent, including over‐the‐counter agents (6.5%), prescription oral medications (6.1%), and prescription topical treatments (4.9%), while hormonal therapy was least reported (2.3%). Notably, 23.7% used multiple strategies, reflecting combined management practices. Detailed responses (see Table S6) showed that 47.6% of students with acne practiced self‐treatment. Most respondents (56.0%) reported not using specific remedies, whereas herbal or natural products such as neem, turmeric, and *aloe vera* were most common (13.9%). Commercial products, including face washes and cosmetic creams, were used by 7.1%. A striking 85.8% reported no product use, while 4% acknowledged various treatments. Among these, both medical products (topical retinoids, benzoyl peroxide, antibiotics) and herbal/natural remedies were reported by about 2% each, and a small proportion mentioned lifestyle modifications such as dietary changes, hydration, and adequate sleep.

## 4 Discussion

This study aimed to assess the prevalence of acne and its impact on quality of life, social appearance anxiety and treatment practices among young adults in Rangpur District, Bangladesh. Our findings indicate a substantial prevalence of acne (47.6%) among the study population, with various demographic, lifestyle and psychosocial factors significantly associated with the condition, consistent with previous studies [7, 9, 20, 21, 22, 23]. Acne remains a common but complex dermatological condition with considerable physical and psychosocial consequences worldwide [24]. The present study sought to explore the predisposing factors, lifestyle behaviors and self‐treatment practices related to acne among university and medical students [9]. The observed prevalence of acne aligns with global reports, ranging from 34.4% in Bangladesh [25] to 97.9% among Saudi female medical students [26, 27, 28], with intermediate rates reported in other countries, including Malaysia (68.1%) [29], India (66.6%) [30], Portugal (62.2%) [31] and Pakistan (55.9%) [21]. In China, moderate‐to‐severe acne has been reported in 10.4% of students [32]. In our study, female participants were more affected than males (45.6%, *p* < 0.001), consistent with global evidence indicating higher acne prevalence among women, potentially due to hormonal influences and the use of comedogenic cosmetics [33, 34]. Gender differences have also been shown to influence psychosocial outcomes, with women experiencing greater emotional and social impairment due to acne [35]. Age was another significant factor, with participants older than 23 years reporting higher prevalence (31.4%) compared to younger groups. This finding is consistent with previous studies in Europe and elsewhere, which have reported self‐reported acne prevalence ranging from 61% to 93% among adolescents and young adults [36, 37, 38]. Family history emerged as a strong predictor of acne, supporting the role of genetic susceptibility in acne pathogenesis [39, 40, 41, 42, 43]. Among lifestyle factors, dietary perceptions showed a significant association with acne which is consistent with studies linking high‐glycemic‐load or dairy‐rich diets to increased acne severity [41]. Smoking and alcohol consumption were also associated with higher acne risk. Smoking may exacerbate acne through oxidative stress and altered sebum composition, while alcohol can influence hormonal regulation and systemic inflammation, potentially aggravating lesions [42, 43]. However, the literature on these associations is mixed, with some studies reporting positive, negative, or null relationships [44, 45, 46, 47]. Most participants in this study experienced mild to moderate acne, consistent with population‐based studies indicating that less severe forms predominate in community settings [4, 48]. Importantly, acne severity was significantly associated with impaired quality of life as measured by the DLQI (*p* = 0.01), highlighting the substantial burden of acne on daily functioning and well‐being. Higher acne severity was also linked with increased social appearance anxiety, reflecting heightened concerns related to self‐image and social interactions [49, 50, 51, 52]. In contrast, generalized anxiety and depression, as assessed by HADS, were not independently associated with acne severity, suggesting that psychological distress may not increase linearly with disease severity, a finding reported in several international studies [20, 53, 54]. Notably, the treatment and management findings revealed considerable gaps in professional care‐seeking and adherence. Only one‐third of affected participants consulted a dermatologist, while the majority relied on self‐medication, home remedies, or no treatment at all [55, 56]. Poor adherence to treatment regimens was common and the frequent use of herbal or cosmetic products highlights a preference for informal management strategies [57]. These patterns may reflect limited access to dermatological services, lack of awareness regarding evidence‐based treatments, or underestimation of acne as a medical condition. Similar trends of self‐treatment and low professional consultation rates have been reported in other low‐ and middle‐income settings, raising concerns about delayed care, inappropriate medication use, and suboptimal outcomes [58].

Overall, these findings underscore that acne exerts a considerable impact on quality of life and social appearance concerns, with demographic, familial, lifestyle, and behavioral factors contributing to its prevalence and management. Although generalized anxiety and depression were not independently associated with acne severity in this study, the significant impairment in quality of life and heightened social appearance anxiety highlight the need for comprehensive, patient‐centered care. From a policy perspective, these results support the integration of dermatological services with mental health support within university health facilities to facilitate early identification of psychosocial distress, improve treatment adherence, and ensure holistic management of acne among young adults. In addition, the high reliance on self‐medication and home remedies emphasizes the importance of awareness and educational programs within academic institutions to discourage unsafe treatment practices and promote evidence‐based care. Given the influence of sociocultural norms on body image and appearance‐related concerns, further research is warranted to explore how cultural contexts shape social appearance anxiety in young adults with acne. Finally, comparative studies involving diverse geographic regions and populations are recommended to enhance the generalizability of these findings and to better inform context‐specific prevention and intervention strategies.

## 5 Limitation of the Study

This study has several limitations. First, the cross‐sectional design prevents establishing causal relationships between the identified factors and acne occurrence. Future longitudinal studies are required to establish causal relationships and evaluate lifestyle‐based interventions. Second, self‐reported data may have introduced recall or reporting bias, particularly regarding lifestyle practices and psychological measures. Third, the study was geographically restricted to university students in northern Bangladesh, which may limit the generalizability of the findings to other regions, age groups, or populations; multicenter and regionally diverse studies are therefore recommended. In addition, although validated instruments were used, further discussion of cultural adaptation of psychosocial scales may strengthen interpretation. Finally, the effectiveness of different treatment practices was not evaluated, and potential confounders such as stress, sleep quality, and detailed dietary patterns were not comprehensively assessed, warranting further investigation.

## 6 Conclusion

Acne vulgaris is highly prevalent among young adults in northern Bangladesh, with nearly half of the surveyed students reporting current symptoms. Female participants, family history, rural residence, smoking, alcohol consumption, and perceived dietary influences emerged as significant predictors. Beyond its physical effects, acne imposes a substantial psychosocial burden, negatively affecting quality of life and social appearance, although links with generalized anxiety and depression were less pronounced. These findings highlight the multifactorial nature of acne, reflecting genetic, lifestyle, and psychosocial influences. These findings underscore the need for university health services to integrate dermatological care with mental health support to address both the physical and psychosocial burden of acne. Given its prevalence and impact, acne should be recognized as more than a cosmetic issue and addressed in student health programs through evidence‐based management, lifestyle interventions, and psychosocial support. Future longitudinal and interventional studies are needed to clarify causal pathways and guide comprehensive prevention and treatment strategies tailored to young adults in Bangladesh and similar populations.

## Author Contributions

All authors contributed significantly to the study. **Tajin Ahmed Jisa:** contributed to study design, data collection and preprocessing, statistical analysis and drafting of the manuscript. **Md. Tahalil Islam Rahat and Most. Shermin Akter Sumi:** involved in literature review, data collection, statistical analysis and result interpretation. **Nourin Sultana and Jibon Kumar Sarker:** assisted with coding, visualization, validation of results, and manuscript editing. **Mifta Nurejannath:** contributed to data acquisition, software and critical review of the manuscript. **Md. Kaderi Kibria:** conceived and supervised the study, provided expertise in machine learning and biostatistics, guided interpretation, and finalized the manuscript.

## Funding

The authors have nothing to report.

## Ethics Statement

The authors confirm adherence to the ethical policies outlined in the journal's author guidelines. Ethical approval was granted by the Institutional Ethical Review Board of the Institute of Research and Technology (IRT), Hajee Mohammad Danesh Science and Technology University (HSTU), under approval number PAS/2025/03.

## Consent

Written informed consent was obtained from all participants prior to data collection. The study objectives and procedures as well as the potential implications were clearly explained to ensure voluntary participation.

## Conflicts of Interest

The authors declare no conflicts of interest.

## Supporting information


**Data S1:** supporting Information.

**Data S2:** supporting Information.

**Table S1:** Assessment of acne‐related lifestyle and mental health parameters.
**Table S2:**; Association between acne prevalence and socio‐demographic, lifestyle and psychosocial factors among participants.
**Table S3:**; Effect of DLQI on acne vulgaris.
**Table S4:**; Effect of SAAS on acne vulgaris.
**Table S5:**; Effect of HADS on acne vulgaris.
**Table S6:**; Frequency distribution of acne remedies and products used among respondents.

## Data Availability

The data that support the findings of this study are available from the corresponding author upon reasonable request.

## References

[1] “Reportlinker Global Acne Market Report for 2016–2026,” (2018).

[2] Y. Assefa , S. Yadav , M. K. Mondal , et al., “Crop Diversification in Rice‐Based Systems in the Polders of Bangladesh: Yield Stability, Profitability, and Associated Risk,” Agricultural Systems 187 (2021): 283–288, 10.1016/j.agsy.2020.102986.

[3] T. Sultana , “Evaluation of Severity in Patients of Acne Vulgaris by Global Acne Grading System in Bangladesh,” Clinical Pathology Research Journal 1 (2017): 28, 10.23880/cprj-16000105.

[4] J. K. L. Tan and K. Bhate , “A Global Perspective on the Epidemiology of Acne,” British Journal of Dermatology 172 (2015): 3–12. 10.1111/bjd.13462 25597339

[5] M. R. Đurović , M. Đurović , J. Janković , and S. Janković , “Quality of Life in Montenegrin Pupils With Acne,” PLoS One 16 (2021): 1–10, 10.1371/journal.pone.0250155. PMC8049225 33857237

[6] S. Kumar , R. Singh , S. Kaur , and B. B. Mahajan , “Psychosocial Impact of Acne on Quality of Life in North India: A Hospital‐Based Cross‐Sectional Study,” Journal of Pakistan Association of Dermatologists 26 (2016): 35–40.

[7] T. Noor , A. S. M. Morshed , A. Ahmed , et al., “Association of Depression, Anxiety and Stress in Acne Vulgaris in Bangladesh,” Journal of Pakistan Association of Dermatologists 33 (2023): 424–428.

[8] J. A. Cotterill and W. J. Cunliffe , “Suicide in Dermatological Patients,” British Journal of Dermatology 137 (1997): 246–250, 10.1046/j.1365-2133.1997.18131897.x. 9292074

[9] D. Dabash , H. Salahat , S. Awawdeh , et al., “Prevalence of Acne and Its Impact on Quality of Life and Practices Regarding Self‐Treatment Among Medical Students,” Scientific Reports 14 (2024): 1–12, 10.1038/s41598-024-55094-6. 38388743 PMC10883973

[10] H. P. M. Gollnick , A. Y. Finlay , and N. Shear , “Can we Define Acne as a Chronic Disease? If So, How and When?,” American Journal of Clinical Dermatology 9 (2008): 279–284. 18717602 10.2165/00128071-200809050-00001

[11] J. E. A. Common , J. N. Barker , and M. A. M. van Steensel , “What Does Acne Genetics Teach us About Disease Pathogenesis?,” British Journal of Dermatology 181 (2019): 665–676. 30854635 10.1111/bjd.17721

[12] M. Sachdeva , J. Tan , J. Lim , M. Kim , I. Nadeem , and R. Bismil , “The Prevalence, Risk Factors, and Psychosocial Impacts of Acne Vulgaris in Medical Students: A Literature Review,” International Journal of Dermatology 60 (2021): 792–798. 33128470 10.1111/ijd.15280

[13] Z. Alrabiah , A. Arafah , M. U. Rehman , et al., “Prevalence and Self‐Medication for Acne Among Students of Health‐Related Science Colleges at King Saud University in Riyadh Region Saudi Arabia,” Medicine 59 (2023): 52, 10.3390/medicina59010052. PMC9863386 36676676

[14] W. Al‐Kubaisy , N. N. Abdullah , S. M. Kahn , and M. Zia , “Sociodemographic Characteristics of Acne Among University Students in Damascus, Syria,” Epidemiology Research International 2014 (2014): 1–4, 10.1155/2014/974019.

[15] L. T. Huei , N. S. F. B. Badaruddin , F. S. Ying , M. A. Bujang , and P. Muniandy , “Prevalence and Psychosocial Impact of Acne Vulgaris Among High School and University Students in Sarawak, Malaysia,” Medical Journal of Malaysia 77 (2022): 446–453. 35902934

[16] J. H. Yang , E. J. Hwang , J. Moon , et al., “Clinical Efficacy of Herbal Extracts in Treatment of Mild to Moderate Acne Vulgaris: An 8‐Week, Double‐Blinded, Randomized, Controlled Trial,” Journal of Dermatological Treatment 32, no. 3 (2021): 297–301, 10.1080/09546634.2019.1657792. Epub 2019 Oct 16. 31424962

[17] B. Koria , S. Bhimani , R. H. Patel , et al., “Dermatology Life Quality Index (DLQI) and Its Determinants Among Patients Attending Tertiary Care Hospitals, in the Western Part of India,” SSR Institute of International Journal of Life Sciences 10 (2024): 6520–6525, 10.21276/ssr-iijls.2024.10.6.24.

[18] M. Goodarzi , M. Noori , M. Aslzakerlighvan , and I. Abasi , “Persian Version of Social Appearance Anxiety Scale: A Psychometric Evaluation,” Iran. J. Psychiatry Behav Sci 15 (2021): 1–6, 10.5812/ijpbs.113164.

[19] A. S. Zigmond , I. Michopoulos , A. Douzenis , et al., “Hospital Anxiety and Depression Scale (HADS): Validation in a Greek General Hospital Sample,” Annals of General Psychiatry 7 (2008): 4–370. 18325093 10.1186/1744-859X-7-4 PMC2276214

[20] F. Asad , A. Qadir , and M. Luqman Ahmed , “Anxiety and Depression in Patients With Acne Vulgaris,” Journal of Pakistan Association of Dermatologists 12 (2002): 69–72.

[21] O. Babar and A. Mobeen , “Prevalence and Psychological Impact of Acne Vulgaris in Female Undergraduate Medical Students of Rawalpindi and Islamabad, Pakistan,” Cureus 11 (2019): e5722, 10.7759/cureus.5722. 31720190 PMC6823076

[22] T. Tasneem , A. Begum , M. R. K. Chowdhury , et al., “Effects of Acne Severity and Acne‐Related Quality of Life on Depressive Symptoms Among Adolescents and Young Adults: A Cross‐Sectional Study in Bangladesh,” Frontiers in Psychology 14 (2023): 1–9, 10.3389/fpsyg.2023.1153101. PMC10405733 37554134

[23] S. Rayapureddy , T. Benerji , M. Kodali , R. Pallekona , H. Enamurthy , and M. Ravi Kumar , “Anxiety and Depression in Patients With Acne Vulgaris at Tertiary Care Hospital: A Cross‐Sectional Study,” Journal of Dr. NTR University of Health Sciences 11 (2022): 351, 10.4103/jdrntruhs.jdrntruhs_88_22.

[24] E. Tasoula , S. Gregoriou , J. Chalikias , et al., “The Impact of Acne Vulgaris on Quality of Life and Psychic Health in Young Adolescents in Greece: Results of a Population Survey,” Anais Brasileiros de Dermatologia 87 (2012): 862–869, 10.1590/s0365-05962012000600007. 23197205 PMC3699905

[25] M. Saizuddin , M. S. Hasan , S. K. Sen , et al., “A Study on Factors in Fluencing Acne Among the Medical Students of Bangladesh,” Med. Today 29 (2017): 30–32, 10.3329/medtoday.v29i2.34623.

[26] S. Zari and D. Alrahmani , “The Association Between Stress and Acne Among Female Medical Students in Jeddah, Saudi Arabia,” Clinical, Cosmetic and Investigational Dermatology 10 (2017): 503–506, 10.2147/CCID.S148499. 29255370 PMC5722010

[27] Z. Allayali , B. N. Asseri , N. I. AlNodali , R. N. M. Alhunaki , and S. F. G. Algoblan , “Assessment of Prevalence, Knowledge, Attitude, and Psychosocial Impact of Acne Vulgaris Among Medical Students in Saudi Arabia,” Journal of Clinical & Experimental Dermatology Research 8, no. 404 (2017): 1–7, 10.4172/2155-9554.1000404.

[28] A. Alajlan , Y. A. Al Turki , Y. AlHazzani , et al., “Prevalence, Level of Knowledge and Lifestyle Association With Acne Vulgaris Among Medical Students,” Journal of Dermatology & Dermatologic Surgery 21 (2017): 58–61, 10.1016/j.jdds.2017.01.001.

[29] L. Muthupalaniappen , H. C. Tan , J. W. D. Puah , M. Apipi , A. E. Sohami , and N. F. Mahat , “Acne Disability, Self Management and Help‐Seeking Behaviour Among Medical Students,” Medical & Health (Universiti Kebangsaan Malaysia) 10 (2015): 1.

[30] N. Joseph , G. Kumar , and M. Nelliyanil , “Skin Diseases and Conditions Among Students of a Medical College in Southern India,” Indian Dermatology Online Journal 5 (2014): 19–24, 10.4103/2229-5178.126023. 24616849 PMC3937480

[31] G. Gonãalves , J. M. Amado , M. E. Matos , and A. Massa , “The Prevalence of Acne Among a Group of Portuguese Medical Students,” Journal of the European Academy of Dermatology and Venereology 26 (2012): 514–517, 10.1111/j.1468-3083.2011.04080.x. 21518023

[32] L. Zhu , M. X. Shen , E. Samran , et al., “Prevalence of Acne in Chinese College Students and Its Associations With Social Determinants and Quality of Life: A Population‐Based Cross‐Sectional Study,” Chinese Medical Journal 134 (2021): 1239–1241, 10.1097/CM9.0000000000001292. 34018999 PMC8143762

[33] N. F. Goodman , R. H. Cobin , W. Futterweit , J. S. Glueck , R. S. Legro , and E. Carmina , “American Association of Clinical Endocrinologists, American College of Endocrinology, and Androgen Excess and Pcos Society Disease State Clinical Review: Guide to the Best Practices in the Evaluation and Treatment of Polycystic Ovary Syndrome ‐ Part 2,” Endocrine Practice 21 (2015): 1415–1426. 26642102 10.4158/EP15748.DSCPT2

[34] G. K. Pruthi and N. Babu , “Physical and Psychosocial Impact of Acne in Adult Females,” Indian Journal of Dermatology 57 (2012): 26–29, 10.4103/0019-5154.92672. 22470204 PMC3312651

[35] L. Misery , P. Wolkenstein , J. M. Amici , et al., “Consequences of Acne on Stress, Fatigue, Sleep Disorders and Sexual Activity: A Population‐Based Study,” Acta Dermato‐Venereologica 95 (2015): 485–488, 10.2340/00015555-1998. 25365961

[36] A. Di Landro , S. Cazzaniga , F. Parazzini , et al., “Family History, Body Mass Index, Selected Dietary Factors, Menstrual History, and Risk of Moderate to Severe Acne in Adolescents and Young Adults,” Journal of the American Academy of Dermatology 67 (2012): 1129–1135, 10.1016/j.jaad.2012.02.018. 22386050

[37] S. X. Xu , H. L. Wang , X. Fan , et al., “The Familial Risk of Acne Vulgaris in Chinese Hans ‐ A Case‐Control Study,” Journal of the European Academy of Dermatology and Venereology 21 (2007): 602–605, 10.1111/j.1468-3083.2006.02022.x. 17447973

[38] E. B. Cho , J. M. Ha , E. J. Park , K. H. Kim , and K. J. Kim , “Heredity of Acne in Korean Patients,” Journal of Dermatology 41 (2014): 915–917, 10.1111/1346-8138.12606. 25200671

[39] V. Bataille , H. Snieder , A. J. MacGregor , P. Sasieni , and T. D. Spector , “The Influence of Genetics and Environmental Factors in the Pathogenesis of Acne: A Twin Study of Acne in Women,” Journal of Investigative Dermatology 119 (2002): 1317–1322, 10.1046/j.1523-1747.2002.19621.x. 12485434

[40] B. Dréno , C. Jean‐Decoster , and V. Georgescu , “Profile of Patients With Mild‐To‐Moderate Acne in Europe: A Survey,” European Journal of Dermatology 26 (2016): 177–184, 10.1684/ejd.2015.2722. 27032481

[41] M. Ulvestad , E. Bjertness , F. Dalgard , and J. A. Halvorsen , “Acne and Dairy Products in Adolescence: Results From a Norwegian Longitudinal Study,” Journal of the European Academy of Dermatology and Venereology 31 (2017): 530–535, 10.1111/jdv.13835. 27422392

[42] T. Francesco , R. Filippo , R. Giuseppe , and R. Antonio , “Compound in Monotherapy for Three Months in Patients With LUTS: Observational Study on Improvement of Urinary Symptoms and Sexual Function in Men,” Health 11 Tradamixina TP (2019): 621–629, 10.4236/health.2019.116052.

[43] C. Kristeen , “Does Alcohol Cause Acne?,” Health (2018).

[44] T. Schäfer , A. Nienhaus , D. Vieluf , J. Berger , and J. Ring , “Epidemiology of Acne in the General Population: The Risk of Smoking,” British Journal of Dermatology 145 (2001): 100–104, 10.1046/j.1365-2133.2001.04290.x. 11453915

[45] A. A. T. Chuh , V. Zawar , W. C. W. Wong , and A. Lee , “The Association of Smoking and Acne in Men in Hong Kong and in India: A Retrospective Case‐Control Study in Primary Care Settings,” Clinical and Experimental Dermatology 29 (2004): 597–599, 10.1111/j.1365-2230.2004.01646.x. 15550130

[46] A. Firooz , R. Sarhangnejad , S. M. Davoudi , and M. Nassiri‐Kashani , “Acne and Smoking: Is There a Relationship?,” BMC Dermatology 5 (2005): 11–13, 10.1186/1471-5945-5-2. 15790395 PMC1079805

[47] I. Klaz , I. Kochba , T. Shohat , S. Zarka , and S. Brenner , “Severe Acne Vulgaris and Tobacco Smoking in Young Men,” Journal of Investigative Dermatology 126 (2006): 1749–1752, 10.1038/sj.jid.5700326. 16645586

[48] K. Pakornphadungsit , S. Harnchoowong , and P. Wattanakrai , “Evaluation of an Acne Severity Grading Self‐Assessment System Suitable for the Thai Population – A Pilot Study,” Clinical, Cosmetic and Investigational Dermatology 16 (2023): 3171–3179, 10.2147/CCID.S427648. 37941850 PMC10629409

[49] B. Dreno , J. M. Amici , A. L. Demessantflavigny , et al., “The Impact of Acne, Atopic Dermatitis, Skin Toxicities and Scars on Quality of Life and the Importance of a Holistic Treatment Approach,” Clinical, Cosmetic and Investigational Dermatology 14 (2021): 623–632, 10.2147/CCID.S315846. 34163201 PMC8213955

[50] S. Purdy and D. de Berker , “Acne Vulgaris,” BMJ Clinical Evidence 2011 (2011): 1714. PMC3275168 21477388

[51] A. Kokandi , “Evaluation of Acne Quality of Life and Clinical Severity in Acne Female Adults,” Dermatology Research and Practice 2010 (2010): 16–18, 10.1155/2010/410809. PMC2913789 20706683

[52] “Acne Prevalence and beyond: Acne Disability and Its Predictive Factors among Chinese Late Adolescents in Hong Kong,” 10.1111/j.1365-2230.2009.03340.x. 19486044

[53] P. Wolkenstein , A. Machovcová , J. C. Szepietowski , D. Tennstedt , S. Veraldi , and A. Delarue , “Acne Prevalence and Associations With Lifestyle: A Cross‐Sectional Online Survey of Adolescents/Young Adults in 7 European Countries,” Journal of the European Academy of Dermatology and Venereology 32 (2018): 298–306, 10.1111/jdv.14475. 28707712

[54] A. Tatliparmak , B. Aksoy , and A. S. Karadağ , “Which Quality of Life Scale Should be Used to Evaluate Acne Vulgaris Patients? CADI or DLQI? A Prospective Study,” Archives of Clinical and Experimental Medicine 4 (2019): 90–93, 10.25000/acem.578444.

[55] M. Albeshri , “Self‐Acne Treatment Among Medical Field Students at Qassim University,” Cureus 17 (2025): e91820, 10.7759/cureus.91820. 41069919 PMC12505412

[56] A. Batalla , A. E. Martínez‐Santos , S. Braña Balige , et al., “Dermatology Self‐Medication in Nursing Students and Professionals: A Multicentre Study,” Health 12 (2024): 1–12, 10.3390/healthcare12020258. PMC10815575 38275538

[57] S. M. Ali , R. T. Brodell , R. Balkrishnan , and S. R. Feldman , “Poor Adherence to Treatments: A Fundamental Principle of Dermatology,” Archives of Dermatology 143 (2007): 912–915, 10.1001/archderm.143.7.912. 17638737

[58] H. Al‐Omrani , M. K. Marwah , R. Al‐Whaib , M. Mekkawy , and H. Shokr , “Patterns of Drug Utilization and Self‐Medication Practices: A Cross Sectional Study,” Pharmacy 11 (2023): 183, 10.3390/pharmacy11060183. 38133458 PMC10747327

