# Supplementary material for: Prevalence of Acne and Its Impact on Quality of Life, Social Appearance Anxiety and Treatment Practices Among Young Adults
Source: J Cosmet Dermatol. 2026 Jan 5;25(1):e70654. doi: 10.1111/jocd.70654 (PMC12766362; doi:10.1111/jocd.70654)
Supplement: Supplementary file 1 — Data S1: supporting Information. [file JOCD-25-e70654-s003.docx]

**Written Informed Consent Form**

**Study Title:** *Prevalence of Acne and Its Impact on Quality of Life, Social Appearance Anxiety, and Treatment Practices among Young Adults*

**Principal Investigator:** Md. Kaderi Kibria

**Institution:** Department of Statistics, Hajee Mohammad Danesh Science and Technology University, Dinajpur-5200

**Purpose of the Study**

You are being invited to participate in a research study that seeks to assess the prevalence of acne among young adults and to understand how it affects quality of life, social appearance anxiety, and treatment practices.

**Procedures**

- You will be asked to complete a questionnaire that includes questions about your demographic information, acne history, its impact on your daily life, and your treatment practices.
- The survey will take approximately 15–20 minutes to complete.

**Voluntary Participation**

- Participation is completely voluntary.
- You may choose not to answer any question or withdraw from the study at any time without penalty.

**Risks and Benefits**

- There are no significant risks anticipated in this study.
- Some questions may cause mild discomfort when reflecting on personal experiences.
- There are no direct benefits, but your participation will contribute to valuable knowledge that may help improve acne management and support for young adults.

**Confidentiality**

- All responses will remain confidential and used solely for research purposes.
- Your identity will not be disclosed in any reports or publications.
- Data will be stored securely and accessed only by the research team.

**Contact Information**

If you have any questions about this study, please contact:
Phone: 01706 719250

Email: kibria.stt@tch.hstu.ac.bd

**Consent Statement**

I have read (or had read to me) the information above. I understand the purpose, procedures, risks, and benefits of this study. I voluntarily agree to participate.

Participant’s Name: ___________________________
Signature/Thumbprint: _________________________
Date: _______________

Researcher’s Name: ___________________________
Signature: _________________________
Date: _______________

**Questionnaire**

Prevalence of Acne and Its Impact on Quality of Life, Social Appearance Anxiety, and Treatment Practices among Young Adults

**A. Demographic Information**

1. Name:
2. Institute name:
3. Age : a.<20 b.20–23 c.>23
4. Gender: a. Male b. Female
5. Living environment: a. Urban b. Rural c. Suburban
6. Marital status: a. Unmarried b. Married
7. Education: a. First year b. Second year c. Third year d. Fourth year e. Fifth year f. Graduated
8. Family income / Socioeconomic status: a. Rich b. Middle c. Poor

**B. Acne Characteristics**

1. How many hours do you sleep on average per night?
2. Do you smoke? a. Yes b. No
3. Do you consume alcohol? a. Yes b. No
4. Have any family history facing acne vulgaris? a. Yes b. No
5. Do you experience acne? a. Yes b. No
6. How severe is your acne on the face? a. Mild b. Moderate c. Severe d. Very severe
7. How long have you experienced acne? a . Less than 6 month’s b. 6 months to 1 year c. 1-3 years d. More than 3 years e. Not experienced

**C. Acne severity measurement scale (Global Acne Grading System (GAGS))**

**(**0 = None 1 = Comedones 2 = Papules 3 = Pustules 4 = Nodules)

| **SN** | **Region** | **Factor** | **Most Severe Lesion Present** |
| --- | --- | --- | --- |
| 1. | Forehead | 2 | □ None □ Comedones □ Papules □ Pustules  □ Nodules |
| 2. | Right Cheek | 2 | □ None □ Comedones □ Papules □ Pustules  □ Nodules |
| 3. | Left Cheek | 2 | □ None □ Comedones □ Papules □ Pustules  □ Nodules |
| 4. | Nose | 1 | □ None □ Comedones □ Papules □ Pustules  □ Nodules |
| 5. | Chin | 1 | □ None □ Comedones □ Papules □ Pustules  □ Nodules |
| 6. | Chest/Back | 3 | □ None □ Comedones □ Papules □ Pustules  □ Nodules |

**D. Quality of Life Assessment (using DLQI scale)**

(0 = Not at all, 1 = A little, 2 = A lot, 3 = Very much)

| Questions | | 0 | 1 | 2 | 3 |
| --- | --- | --- | --- | --- | --- |
|  | Over the last week, how itchy, painful, sore, or stinging has your skin been? |  |  |  |  |
|  | Over the last week, how embarrassed or self-conscious have you been because of your skin? |  |  |  |  |
|  | Over the last week, how much has your skin interfered with your daily activities (shopping, work, or home care)? |  |  |  |  |
|  | Over the last week, how much has your skin influenced the clothes you wear? |  |  |  |  |
|  | Over the last week, how much has your skin affected any social or leisure activities? |  |  |  |  |
|  | Over the last week, how much has your skin made it difficult for you to do sports or other physical activities? |  |  |  |  |
|  | Over the last week, has your skin prevented you from working or studying? If not, how much has it been a problem at work or school |  |  |  |  |
|  | Over the last week, how much has your skin created problems with your partner or any close relationships |  |  |  |  |
|  | Over the last week, how much has the treatment for your skin been a problem (messy, time-consuming)? |  |  |  |  |
|  | Over the last week, how much has your skin made you feel emotionally distressed? |  |  |  |  |

**E. Social Appearance Anxiety (using SAAS scale)**

(Strongly=0, Disagree=1, Disagree=2, Neutral Agree =3, Strongly Agree=4)

| Questions | | 0 | 1 | 2 | 3 | 4 |
| --- | --- | --- | --- | --- | --- | --- |
|  | I feel comfortable with the way I appear to others. |  |  |  |  |  |
|  | I feel nervous when having my picture taken. |  |  |  |  |  |
|  | I get tense when it is obvious people are looking at me. |  |  |  |  |  |
|  | I am concerned people would not like me because of the way I look. |  |  |  |  |  |
|  | I worry that others talk about flaws in my appearance when I am not around. |  |  |  |  |  |
|  | I am concerned people will find me unappealing because of my appearance. |  |  |  |  |  |
|  | I am afraid that people find me unattractive. |  |  |  |  |  |
|  | I worry that my appearance will make life more difficult for me. |  |  |  |  |  |
|  | I am concerned that I have missed out on opportunities because of my appearance. |  |  |  |  |  |
|  | I get nervous when talking to people because of the way I look. |  |  |  |  |  |
|  | I feel anxious when other people say something about my appearance. |  |  |  |  |  |
|  | I am frequently afraid I would not meet others’ standards of how I should look. |  |  |  |  |  |
|  | I worry people will judge the way I look negatively. |  |  |  |  |  |
|  | I am uncomfortable when I think others are noticing flaws in my appearance. |  |  |  |  |  |
|  | I worry that a romantic partner will/would leave me because of my appearance. |  |  |  |  |  |
|  | I am concerned that people think I am not good looking. |  |  |  |  |  |

**F. Psychological Impact** (Not at all=0, Sometimes=1, Often=2, Always=3)

1. I feel tense or nervousness.
2. I still enjoy the things I used to enjoy.
3. I get a sort of frightened feeling as if something awful is about to happen
4. I feel as if I am slowed down
5. Worrying thoughts go through my mind
6. I can laugh and see the funny side of things
7. I can sit at ease and feel relaxed
8. I feel cheerful
9. I get a sort of frightened feeling like 'butterflies' in the stomach
10. I have lost interest in my appearance
11. I feel restless as I have to be on the move
12. I look forward with enjoyment to things
13. I get sudden feelings of panic
14. I can enjoy a good book or radio or TV program

**G. Treatment and Management**

1. Have you consulted a dermatologist for your acne? a. Yes b. No
2. Which types of acne treatments have you used? (Select all that apply) a. Over-the-counter treatments (e.g., benzoyl peroxide, salicylic acid) b. Prescription oral medication (e.g., antibiotics, isotretinoin) c. Prescription topical treatments (e.g., retinoids) d. Hormonal treatments (e.g., birth control, spironolactone)e. Natural/home remedies
3. How often do you follow your acne treatment routine? a. Always b. Often c. Sometimes d. Rarely c. Never
4. Have you tried any home remedies or herbal treatments for acne? (Yes/No)
5. Specify the remedies used: ______________
6. Do you self-medicate for acne? (Yes/No)
7. If yes, what products or methods do you use?

<><><><><><> Thank you for your co-operation <><><><><>
