# Supplementary material for: Prevalence of Acne and Its Impact on Quality of Life, Social Appearance Anxiety and Treatment Practices Among Young Adults
Source: J Cosmet Dermatol. 2026 Jan 5;25(1):e70654. doi: 10.1111/jocd.70654 (PMC12766362; doi:10.1111/jocd.70654)
Supplement: Supplementary file 2 — Data S2: supporting Information. [file JOCD-25-e70654-s001.docx]

**2.5.2 Acne severity measurement scales**

The GAGS is a quantitative scoring system to assess acne severity. It was first developed by Doshi and colleagues in 1997. The total severity score is derived from summation of six regional sub scores. Each is derived by multiplying the factors-2 for forehead, 2 for each check, 1 for nose, 1 for chin, 3 for both chest and back by the most heavily weighted lesion within each region (1 for ≥ one comedone, 2 for ≥ one papule,3 for ≥ one pustule, and 4 for ≥ one nodule). The regional factors were derived from consideration of surface area and distribution and density of pilosebaceous units [1] (Table 1).

Table 1: The Global Acne Grading System.

| Location | Factor X Grade (0-4) = local score | |
| --- | --- | --- |
| Forehead | 2 | [Global score =  0 = None  1-18 = Mild  19-30 = Moderate  31-38 = Severe  > 39 = Very severe] |
| Right cheek | 2 |  |
| Left cheek | 2 |  |
| Nose | 1 |  |
| Chin | 1 |  |
| Chest & upper back 3 | 3 |  |

Grade 0, No lesions; 1 ≥ One comedone; 2 ≥ One papule; 3 ≥ One pustule; 4 ≥ One nodule

**Dermatology Life Quality Index (DLQI)**

The DLQI questionnaire consists of 10 questions encompassing various domains which includes cutaneous symptoms like itching, burning or stinging, degree of embarrassment or self-consciousness, interference with daily activities, influence over the choice of clothing, interference with leisure and social activities, difficulty in performing sporting activities, interference with work or study, difficulty in interpersonal relationships, sexual difficulty and problems due to treatment of the condition. Each domain has 4 possible responses ranging from 0 to 3 (0- not at all, 1- little, 2- lot, 3- very much) and the total possible score is 30. The impact on QoL is based on the final score and is graded as no effect (0-1), small effect (2-5), moderate effect (6-10), very large effect (11-20) and extremely large effect (21-30). Data entry was done on MS Excel and data analysis was performed in SPSS 22 version. The study was approved by the ethical and research committee [2].

**Social Appearance Anxiety Scale (SAAS)**

The Social Appearance Anxiety Scale (SAAS) was created to measure anxiety about being negatively evaluated by others because of one's overall appearance, including body shape. The scale, developed by Hart et al., (2008) and adapted to Turkish by Doğan (2011) (11), consists of 16 items and one dimension [3].

**Hospital Anxiety and Depression Scale (HADS)**

The Hospital Anxiety and Depression Scale (HADS) was applied to assess the levels of anxiety and depression in patients with acne vulgaris. The HADS is a self‑report questionnaire designed to assess the levels of anxiety and depression. It consists of two subscales with seven items each, one measuring anxiety, and another measuring depression. Each item is scored on a 4‑point Likert scale (0–3), the possible scores range from 0 to 21 for each of the two subscales. The HADS manual indicates that a score between 0 to 7 is normal; 8–10, mild; 11–14, moderate; and 15–21, severe [4].

**References**

[1] T. Sultana, “Evaluation of Severity in Patients of Acne Vulgaris by Global Acne Grading System in Bangladesh,” *Clin. Pathol. Res. J.*, vol. 1, no. 1, 2017, doi: 10.23880/cprj-16000105.

[2] S. Sivaramakrishnan and T. Jayakar, “A study on the dermatology life quality index in patients with acne vulgaris,” *Int. J. Res. Dermatology*, vol. 5, no. 4, p. 774, 2019, doi: 10.18203/issn.2455-4529.intjresdermatol20194667.

[3] F. Kurhan, “Social appearance anxiety in patients with contact dermatitis,” *East. J. Med.*, vol. 26, no. 4, pp. 589–594, 2021, doi: 10.5505/ejm.2021.52280.

[4] S. Rayapureddy, T. Benerji, M. Kodali, R. Pallekona, H. Enamurthy, and M. Ravi Kumar, “Anxiety and depression in patients with acne vulgaris at tertiary care hospital: A cross-sectional study,” *J. Dr. NTR Univ. Heal. Sci.*, vol. 11, no. 4, p. 351, 2022, doi: 10.4103/jdrntruhs.jdrntruhs_88_22.
