# Supplementary material for: Prevalence of Acne and Its Impact on Quality of Life, Social Appearance Anxiety and Treatment Practices Among Young Adults
Source: J Cosmet Dermatol. 2026 Jan 5;25(1):e70654. doi: 10.1111/jocd.70654 (PMC12766362; doi:10.1111/jocd.70654)
Supplement: Supplementary file 3 — Table S1: Assessment of acne‐related lifestyle and mental health parameters. Table S2:; Association between acne prevalence and socio‐demographic, lifestyle and psychosocial factors among participants. Table S3:; Effect of DLQI on acne vulgaris. Table S4:; Effect of SAAS on acne vulgaris. Table S5:; Effect of HADS on acne vulgaris. Table S6:; Frequency distribution of acne remedies and products used among respondents. [file JOCD-25-e70654-s002.docx]

Supplementary File

**Table S1:** Assessment of Acne-Related Lifestyle and Mental Health Parameters

| **Variables** | **Frequence (%)** |  | **Variables** | **Frequence (%)** |
| --- | --- | --- | --- | --- |
| Experiencing Acne | |  | Small effect | 160 (34.4) |
| Yes | 465 (47.6) |  | Moderate effect | 38 (29.7) |
| No | 511 (52.4) |  | Very large effect | 108 (23.2) |
| Smoking | |  | Extremely large effect | 29 (6.2) |
| Yes | 182 (18.6) |  | SAAS Impact | |
| No | 794 (81.4) |  | Mild | 180 (38.7) |
| Consuming Alcohol | |  | Moderate | 237 (51.0) |
| Yes | 67 (6.9) |  | Severe | 48 (10.3) |
| No | 909 (93.7) |  | HADS | |
| Dietary habits affect Acne | |  | Normal | 56 (5.7) |
| Yes | 568 (58.2) |  | Mild | 638 (65.4) |
| No | 408 (41.8) |  | Moderate | 255 (26.1) |
| DLQI Impact (for yes) | |  | Severe | 27 (2.8) |
| No effect | 30 (6.5) |  | GAGS Severity | |
|  |  |  | Mild | 503 (51.5) |
|  |  |  | Moderate | 398 (40.8) |
|  |  |  | Severe | 63 (6.5) |
|  | |  | Very Severe | 12 (1.2) |

**Table S2**: Association between acne prevalence and socio-demographic, lifestyle and psychosocial factors among participants.

| **Variable** | **Category** | **Frequency (%)** | **Acne**  **(*n* = 465)** | **Without Acne**  **(*n* = 511)** | ***p*-value** |
| --- | --- | --- | --- | --- | --- |
| Age | <20 | 89 (9.1%) | 38 (8.2%) | 51 (10.0%) |  |
|  | 20–23 | 674 (69.1%) | 281 (60.4%) | 393 (76.9%) |  |
|  | >23 | 213 (21.8%) | 146 (31.4%) | 67 (13.1%) | 0.001 |
| Gender | Male | 592 (60.7%) | 253 (54.4%) | 339 (66.3%) |  |
|  | Female | 384 (39.3%) | 212 (45.6%) | 172 (33.7%) | 0.001 |
| Marital Status | Unmarried | 805 (82.5%) | 362 (77.9%) | 443 (86.7%) |  |
|  | Married | 171 (17.5%) | 103 (22.1%) | 68 (13.3%) | 0.001 |
| Living Environment | Urban | 380 (38.9%) | 185 (39.8%) | 195 (38.2%) |  |
|  | Rural | 452(46.3%) | 209 (44.9%) | 243 (47.6%) |  |
|  | Suburban | 144 (14.8%) | 71 (15.3%) | 73 (14.3%) | 0.711 |
| Sleeping hour | <5 hour | 52 (5.3%) | 29 (6.2%) | 23 (4.5%) |  |
|  | 5–7 hour | 526 (53.9%) | 263 (56.6%) | 263 (51.5%) |  |
|  | 7–9 hour | 344 (35.3%) | 153 (32.9%) | 191 (37.4%) |  |
|  | >9 hour | 54 (5.5%) | 20 (4.3%) | 34 (6.7%) | 0.095 |
| Institute | Brur | 232 (23.8%) | 108 (23.2%) | 124 (24.3%) |  |
|  | Rmc | 159 (16.3%) | 72 (15.5%) | 87 (17.0%) |  |
|  | Dmc | 186 (19.1%) | 80 (17.2%) | 106 (20.7%) |  |
|  | Hstu | 399 (40.9%) | 205 (44.1%) | 194 (38.0%) | 0.231 |
| Education | First year | 505 (51.7%) | 208 (44.7%) | 297 (58.1%) |  |
|  | Secnd year | 187 (19.2%) | 88 (18.9%) | 99 (19.4%) |  |
|  | Third year | 169 (17.3%) | 100 (21.5%) | 69 (13.5%) |  |
|  | Fourth year | 79 (8.1%) | 48 (10.3%) | 31 (6.1%) |  |
|  | Graduated | 22 (2.3%) | 12 (2.6%) | 10 (2.0%) |  |
|  | Fifth year/Masters | 11 (1.1%) | 7 (1.5%) | 4 (0.8%) | 0.001 |
| Family Income | Poor | 124 (12.7%) | 59 (12.7%) | 65 (12.7%) |  |
|  | Middle | 725 (74.3%) | 338 (72.7%) | 387 (75.7%) |  |
|  | Rich | 127 (13.0%) | 68 (14.6%) | 59 (11.5%) | 0.354 |
| Family History of Acne | Yes | 362 (37.1%) | 281 (60.4%) | 81 (15.9%) |  |
|  | No | 614 (62.9%) | 184 (39.6%) | 430 (84.1%) | 0.001 |
| Smoking | Yes | 182 (18.7%) | 117 (25.2%) | 65 (12.7%) |  |
|  | No | 794 (81.3%) | 348 (74.8%) | 446 (87.3%) | 0.001 |
| Consuming Alcohol | Yes | 67 (6.8%) | 53 (11.4%) | 14 (2.7%) |  |
|  | No | 909 (93.2%) | 412 (88.6%) | 497 (97.3%) | 0.001 |
| Dietary Habits | Yes | 568 (58.2%) | 352 (75.7%) | 216 (42.3%) |  |
|  | No | 408 (41.8%) | 113 (24.3%) | 295 (57.7%) | 0.001 |

## Table S3: Effect of DLQI on acne vulgaris.

| **Predictor Variable** | **Odds Ratio (OR)** | ***p*-value** | **95% CI (Lower – Upper)** |
| --- | --- | --- | --- |
| DLQI Impact Ref: Low | | | |
| High | 1.682 | 0.002 | 1.216 – 2.327 |
| Age of the respondent (Ref: <20 years) | | | |
| 20–23 years | 0.615 | 0.080 | 0.357 – 1.060 |
| >23 years | 1.317 | 0.458 | 0.637 – 2.724 |
| Gender (Ref: Female) | | | |
| Male | 0.675 | 0.018 | 0.487 – 0.935 |
| Living Environment (Ref: Urban) | | | |
| Rural | 0.549 | 0.001 | 0.384 – 0.784 |
| Suburban | 0.664 | 0.107 | 0.403 – 1.093 |
| Marital Status (Ref: Unmarried) | | | |
| Married | 1.176 | 0.497 | 0.737 – 1.878 |
| Education (Ref: First year) | | | |
| Second year | 1.219 | 0.779 | 0.305 – 4.874 |
| Third year | 0.661 | 0.576 | 0.155 – 2.823 |
| Fourth year | 0.494 | 0.411 | 0.092 – 2.657 |
| Graduate | 0.930 | 0.919 | 0.229 – 3.777 |
| Fifth year | 0.758 | 0.694 | 0.190 – 3.022 |
| Family income (Ref: Poor) | | | |
| Middle income | 0.627 | 0.045 | 0.389 – 0.945 |
| Upper income | 0.614 | 0.138 | 0.322 – 1.169 |
| Belief that diet affects acne (Ref: No) | | | |
| Yes | 3.434 | 0.000 | 2.485 – 4.746 |
| Sleep duration (Ref: <5 hours) | | | |
| 5–7 hours | 1.281 | 0.511 | 0.612 – 2.679 |
| 7–9 hours | 1.021 | 0.957 | 0.482 – 2.164 |
| >9 hours | 0.716 | 0.489 | 0.278 – 1.845 |
| Smoking status (Ref: No) | | | |
| Yes | 1.661 | 0.028 | 1.057 – 2.611 |
| Alcohol consumption (Ref: No) | | | |
| Yes | 3.101 | 0.003 | 1.467 – 6.557 |
| Family history of acne (Ref: No) | | | |
| Yes | 4.112 | 0.000 | 2.335 – 6.518 |

## Table S4: Effect of SAAS on acne vulgaris.

| **Predictor Variable** | **Odds Ratio (OR)** | ***p*-value** | **95% CI (Lower – Upper)** |
| --- | --- | --- | --- |
| SAAS Impact Ref: Mild | | | |
| Moderate | 1.136 | 0.450 | 0.816 – 1.582 |
| Severe | 1.355 | 0.236 | 0.820 – 2.238 |
| Age of the respondent (Ref: <20 years) | | | |
| 20–23 years | 0.678 | 0.158 | 0.396 – 1.162 |
| >23 years | 1.378 | 0.388 | 0.665 – 2.857 |
| Gender (Ref: Female) | | | |
| Male | 0.652 | 0.010 | 0.472 – 0.902 |
| Living Environment (Ref: Urban) | | | |
| Rural | 0.516 | 0.000 | 0.362 – 0.733 |
| Suburban | 0.595 | 0.038 | 0.364 – 0.972 |
| Marital Status (Ref: Unmarried) | | | |
| Married | 1.166 | 0.524 | 0.727 – 1.870 |
| Education (Ref: First year) | | | |
| Second year | 1.062 | 0.933 | 0.260 – 4.334 |
| Third year | 0.596 | 0.492 | 0.136 – 2.613 |
| Fourth year | 0.479 | 0.398 | 0.087 – 2.637 |
| Graduate | 0.931 | 0.921 | 0.224 – 3.856 |
| Fifth year | 0.771 | 0.717 | 0.189 – 3.148 |
| Family income (Ref: Poor) | | | |
| Middle income | 0.611 | 0.043 | 0.379 – 0.984 |
| Upper income | 0.621 | 0.145 | 0.327 – 1.179 |
| Belief that diet affects acne (Ref: No) | | | |
| Yes | 3.465 | 0.000 | 2.511 – 4.780 |
| Sleep duration (Ref: <5 hours) | | | |
| 5–7 hours | 1.200 | 0.629 | 0.572 – 2.518 |
| 7–9 hours | 0.983 | 0.964 | 0.463 – 2.089 |
| >9 hours | 0.760 | 0.569 | 0.295 – 1.957 |
| Smoking status (Ref: No) | | | |
| Yes | 1.699 | 0.021 | 1.083 – 2.668 |
| Alcohol consumption (Ref: No) | | | |
| Yes | 3.501 | 0.001 | 1.671 – 7.335 |
| Family history of acne (Ref: No) | | | |
| Yes | 4.228 | 0.000 | 2.420 – 6.776 |

## Table S5: Effect of HADS on acne vulgaris.

| **Predictor Variable** | **Odds Ratio (OR)** | ***p*-value** | **95% CI (Lower – Upper)** |
| --- | --- | --- | --- |
| HADS Ref: Normal | | | |
| Mild | 1.446 | 0.290 | 0.730 – 2.865 |
| Moderate | 1.317 | 0.458 | 0.636 – 2.728 |
| Severe | 1.873 | 0.268 | 0.618 – 5.682 |
| Age of the respondent (Ref: <20 years) | | | |
| 20–23 years | 0.672 | 0.150 | 0.391 – 1.155 |
| >23 years | 1.400 | 0.354 | 0.677 – 2.895 |
| Gender (Ref: Female) | | | |
| Male | 0.659 | 0.012 | 0.476 – 0.913 |
| Living Environment (Ref: Urban) | | | |
| Rural | 0.514 | 0.000 | 0.362 – 0.732 |
| Suburban | 0.584 | 0.032 | 0.3657– 0.955 |
| Marital Status (Ref: Unmarried) | | | |
| Married | 1.165 | 0.525 | 0.727 – 1.868 |
| Education (Ref: First year) | | | |
| Second year | 1.076 | 0.918 | 0.269 – 4.297 |
| Third year | 0.614 | 0.512 | 0.143 – 2.640 |
| Fourth year | 0.471 | 0.381 | 0.087 – 2.540 |
| Graduate | 0.943 | 0.934 | 0.231 – 3.839 |
| Fifth year | 0.757 | 0.693 | 0.189 – 3.023 |
| Family income (Ref: Poor) | | | |
| Middle income | 0.611 | 0.043 | 0.379 – 0.984 |
| Upper income | 0.633 | 0.163 | 0.333 – 1.204 |
| Belief that diet affects acne (Ref: No) | | | |
| Yes | 3.475 | 0.000 | 2.517 – 4.798 |
| Sleep duration (Ref: <5 hours) | | | |
| 5–7 hours | 1.206 | 0.621 | 0.574 – 2.535 |
| 7–9 hours | 0.990 | 0.979 | 0.464 – 2.112 |
| >9 hours | 0.765 | 0.580 | 0.296 – 1.976 |
| Smoking status (Ref: No) | | | |
| Yes | 1.688 | 0.023 | 1.076 – 2.648 |
| Alcohol consumption (Ref: No) | | | |
| Yes | 3.436 | 0.001 | 1.628 – 7.254 |
| Family history of acne (Ref: No) | | | |
| Yes | 4.227 | 0.000 | 2.461 – 6.833 |

**Table S6:** Frequency distribution of acne remedies and products used among respondents

| Variables | Frequence (%) |  | Variables | Frequence (%) |
| --- | --- | --- | --- | --- |
| Remedies used | |  | Products or methods used | |
| None reported | 547(56.0) |  | None reported | 837(85.8) |
| Herbal and natural remedies (neem,Aloe vera, turmeric, cucumber, honey, Lemon-etc.) | 136(`13.9) |  | Herbal/natural (Neem, Aloe vera, turmeric, etc.) | 20(2.0) |
|  |  |  | Medical products (Antibiotics, topical retinoids, salicylic/benzoyl peroxide, etc.) | 20(2.0) |
|  |  |  | Skincare products (face wash, OXY, ponds, creams, etc.) | 15(1.5) |
| Face wash / cosmetic products(Creams/lotions Blackhead remover / other product) | 70(7.1) |  | Lifestyle modifications (diet, hydration, hygiene) | 6(0.6) |
|  |  |  | Other specified | 78(8.0) |
|  |  |  |  |  |
| Other mixed remedies | 180(18.4) |  |  |  |
